# Creating a sustainable brain health navigator model to improve the diagnostic journey for Alzheimer's disease: the experience of one health care system in California

**DOI:** 10.1002/alz70860_101320

**Published:** 2025-12-23

**Authors:** John Clark, Andrea Snyder, Janet Appel

**Affiliations:** ^1^ Sharp Reese‐Stealy Medical Group, San Diego, CA, USA

## Abstract

**Background:**

The Sharp Rees‐Stealy Medical Centers (SRSMC) serve approximately 350,000 patients annually. Our network includes multiple primary care clinics, specialty care facilities, and hospitals, allowing us to reach a broad spectrum of patients in the Southern California region. SRSMC deliver clinical services including outreach programs in both urban and rural areas ensuring that patients across different geographic locations receive access to healthcare services appropriate for their needs, including cognitive assessments. To address transportation barriers for patients, our available clinical services include in‐home patient visits that commonly use cognitive assessment tools in patients who are homebound and in diverse geographic areas.

**Method:**

The SRSMC pilot will include 130 PCPs that are connected to the program via Population Health. The program includes standardization of workflows to ensure all patients who are at an increased risk for dementia will have regular cognitive assessments. Enhanced provider education will ensure that our staff are well‐equipped to deliver high‐quality care to patients with cognitive needs. By fostering a team‐based approach, we can ensure that patients receive comprehensive care that addresses all aspects of their condition. We will embed technological tools to assist with cognitive assessments into our routine clinical workflows. We will utilize our EHR system to set automated reminders and alerts for follow‐up assessments. Telehealth platforms will be used to reach patients who may have difficulty visiting the clinic in person. We will form multidisciplinary care teams that include primary care physicians, neurologists, gerontologists, psychologists, and care coordinators. Regular team meetings and case reviews will help maintain high standards of care and keep cognitive health a priority.

**Result:**

By integrating cognitive assessments into routine care, we can identify cognitive decline at its earliest stages, which is critical for effective management and treatment. We will provide educational materials, workshops, and support groups to empower patients and caregivers with the knowledge and resources they need to manage brain health effectively.

**Conclusion:**

The Brain Health Navigator Model and other resources of DAC‐SP support the implementation of boundary spanning roles across primary and specialty care, designed for a timely and accurate diagnostic journey across SRSMC and similar health systems.

